# Efficacy of an orally active small-molecule inhibitor of RANKL in bone metastasis

**DOI:** 10.1038/s41413-018-0036-5

**Published:** 2019-01-03

**Authors:** Yuta Nakai, Kazuo Okamoto, Asuka Terashima, Shogo Ehata, Jun Nishida, Takeshi Imamura, Takashi Ono, Hiroshi Takayanagi

**Affiliations:** 1 Department of Immunology, Graduate School of Medicine and Faculty of Medicine, The University of Tokyo, Hongo 7-3-1, Bunkyo-ku, Tokyo Japan; 2 Department of Orthodontic Science, Graduate School of Medical and Dental Sciences, Tokyo Medical and Dental University, Yushima 1-5-45, Bunkyo-ku, Tokyo Japan; 3 Department of Osteoimmunology, Graduate School of Medicine and Faculty of Medicine, The University of Tokyo, Hongo 7-3-1, Bunkyo-ku, Tokyo Japan; 4 Department of Molecular Pathology, Graduate School of Medicine, The University of Tokyo, Hongo 7-3-1, Bunkyo-ku, Tokyo Japan; 5 Department of Molecular Medicine for Pathogenesis, Ehime University Graduate School of Medicine, Shitsukawa, Toon, Ehime Japan

**Keywords:** Bone cancer, Bone

## Abstract

Bone is one of the preferred sites for the metastasis of malignant tumours, such as breast cancer, lung cancer and malignant melanoma. Tumour cells colonizing bone have the capacity to induce the expression of receptor activator of nuclear factor-κB ligand (RANKL), which promotes osteoclast differentiation and activation. Tumour-induced osteoclastic bone resorption leads to a vicious cycle between tumours and bone cells that fuels osteolytic tumour growth, causing bone pain and hypercalcaemia. Furthermore, RANKL contributes to bone metastasis by acting as a chemoattractant to bone for tumour cells that express its receptor, RANK. Thus inhibition of the RANKL–RANK pathway is a promising treatment for bone metastasis, and a human monoclonal anti-RANKL antibody, denosumab, has been used in the clinic. However, orally available drugs targeting RANKL must be developed to increase the therapeutic benefits to patients. Here we report the efficacy of the small-molecule RANKL inhibitor AS2676293 in treating bone metastasis using mouse models. Oral administration of AS2676293 markedly inhibited bone metastasis of human breast cancer cells MDA-MB-231-5a-D-Luc2 as well as tumour-induced osteolysis. AS2676293 suppressed RANKL-mediated tumour migration in the transwell assay and inhibited bone metastasis of the murine cell line B16F10, which is known not to trigger osteoclast activation. Based on the results from this study, RANKL inhibition with a small-molecule compound constitutes a promising therapeutic strategy for treating bone metastasis by inhibiting both osteoclastic bone resorption and tumour migration to bone.

## Oncology: Orally administered RANKL inhibitor promising for treating bone metastasis

Tumor cells colonized in bone can induce expression of a protein called receptor activator of nuclear factor kappa-Β ligand (RANKL), and an orally administered RANKL inhibitor is a promising therapeutic strategy for treating bone metastasis. RANKL contributes to bone metastasis, and so inhibiting RANKL has been regarded as a potential treatment. However, to increase the therapeutic benefit to patients, it is necessary to develop orally administered drugs that target RANKL. A team headed by Hiroshi Takayanagi at The University of Tokyo developed a small-molecule RANKL inhibitor called AS2676293, which proved effective for treating bone metastasis in mouse cell cultures. Oral administration of AS2676293 markedly decreased bone metastasis of human breast cancer cells. The authors believe that inhibiting RANKL with a small-molecule compound holds great promise as a therapeutic agent for bone metastasis.

## Introduction

Bone homeostasis is maintained through osteoblastic bone formation and osteoclastic bone resorption.^1,2^ Receptor activator of nuclear factor-κB ligand (RANKL), a member of the tumour necrosis factor (TNF) family, is an essential cytokine for osteoclastogenesis.^3–5^ RANKL binds to its receptor RANK, which is expressed on osteoclast precursor cells, to induce osteoclast differentiation through the activation of transcription factors, such as nuclear factor of activated T cell c1(NFATc1).^1,6^ Excess osteoclast activity leads to abnormal bone resorption, as observed in a variety of skeletal pathologies in patients with rheumatoid arthritis, periodontal disease, osteoporosis and bone tumours.^1,2,5^

Bone is one of the most common sites of tumour metastasis.^7^ Bone metastasis often results in serious complications, including bone pain, hypercalcaemia, fractures and spinal cord compression, which significantly contribute to a reduced quality of life.^8,9^ Recent advances in cancer therapies have improved patients’ longevity and conversely increased the risk of bone metastasis.

Breast cancer, lung cancer, prostate cancer and malignant melanoma frequently metastasize to bone.^7,10^ Bone metastases of tumour cells are divided into two main types: osteoblastic and osteolytic metastases. Osteoblastic metastasis is nearly always observed in the bone metastasis of prostate cancer, which is the result of osteoblast stimulation by the cancer cells.^11,12^ Factors that are locally produced by the cancer cells, such as bone morphogenetic proteins, insulin-like growth factors (IGFs), fibroblast growth factors, transforming growth factor (TGF)-β and endothelin-1, promote osteoblast proliferation and bone formation.^12^ On the other hand, osteolytic bone metastasis is most often caused by breast cancer and multiple myeloma.^8,10^ Tumour cells stimulate the RANKL expression in bone marrow stromal cells via the production of parathyroid hormone-related peptide, prostaglandin E_2_, interleukin (IL)-6, IL-1β, TNF and epidermal growth factor, resulting in an increase in osteoclastic bone resorption.^10^ Subsequently, growth factors such as TGF-β and IGFs are released from the degraded bone matrices, promoting tumour cell proliferation.^10^ This ‘vicious cycle’ linking the tumour cells, bone marrow stromal cells and osteoclasts underlies the pathogenesis of osteolytic metastasis.^7,8^ Studies using a mouse model of bone metastasis employing the human breast cancer cell line MDA-MB-231, which forms osteolytic metastases, revealed that in vivo neutralization of RANKL with osteoprotegerin (OPG) prevents bone destruction and skeletal tumour growth by suppressing osteoclast activity.^13,14^

RANKL contributes to bone metastasis by not only activating osteoclastic bone resorption but also stimulating the migration of tumour cells to bone.^15–18^ RANK is expressed at high levels on many different epithelial tumour cells that preferentially metastasize to bone, including MDA-MB-231 cells and the murine melanoma cell line B16F10. RANKL acts directly on RANK-expressing tumour cells to induce actin polymerization and increase cell migration.^15^ In a mouse model of bone metastasis using B16F10 cells that do not trigger osteoclast activation, an OPG treatment markedly reduced the tumour burden in the bones, whereas treatment with bisphosphonate had no effect. The OPG treatment did not alter the metastasis of B16F10 cells to other organs, such as the ovaries and adrenal glands, indicating that the chemotactic activity of RANKL is one of the primary causes of the preferential metastasis of RANK-expressing tumour cells to bone.^15^ In support of this hypothesis, the level of RANK expression is reported to be positively correlated with the bone metastatic potential of human primary breast cancer and renal cell carcinomas.^19,20^

Therefore, inhibition of the RANKL–RANK pathway is a therapeutic target for bone metastasis. The fully human monoclonal anti-RANKL antibody denosumab, which inhibits the binding of RANKL to RANK, has been approved as a treatment for osteoporosis and the prevention of skeletal-related events in patients with bone metastasis and giant cell tumours of the bone.^21^ However, since the high cost of therapeutic antibody drugs has an increasing impact on the health-care burden, less expensive alternative approaches such as low-molecular weight inhibitors must be developed. The recently developed inhibitor AS2676293 has been shown to inhibit RANKL-induced osteoclast differentiation of the monocyte/macrophage cell line RAW264.7.^22^ The inhibitory effect of AS2676293 was also determined in vivo using a RANKL-induced osteoporosis model.^22^ As shown in our recent study, the RANKL on T cells stimulates astrocytes in the central nervous system (CNS) during autoimmune encephalomyelitis to promote inflammatory cell infiltration into the CNS. Oral administration of AS2676293 potently inhibits immune cell infiltration,^23^ indicating that AS2676293 is an effective inhibitor of the RANKL–RANK pathway.

In the present study, we investigated the efficacy of the novel RANKL inhibitor AS2676293 as a treatment for bone metastasis using mouse models. Oral administration of AS2676293 decreased the bone metastasis of breast cancer cells and malignant melanoma by inhibiting not only osteoclast activity but also RANKL-induced tumour migration. Based on these findings, inhibition of RANKL with a small-molecule inhibitor is an effective method for inhibiting osteolysis and decreasing the skeletal tumour burden in patients with bone metastasis.

## Results

### AS2676293 inhibits the differentiation of murine bone marrow-derived monocyte/macrophage precursor cells (BMMs) into osteoclasts in vitro

**Fig. 1:**
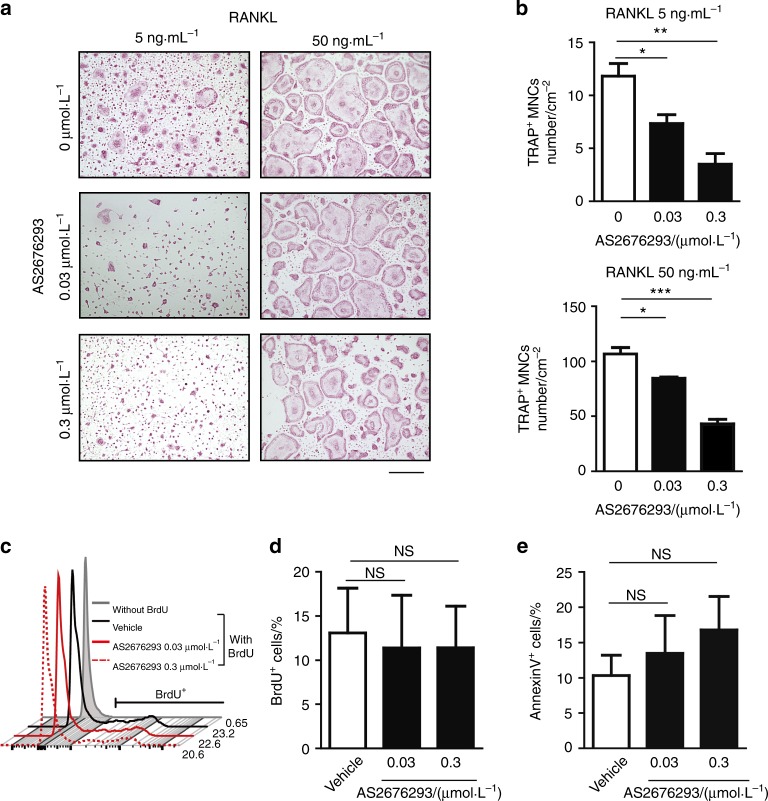
Effect of AS2676293 on osteoclast differentiation in vitro. **a**, **b** Effect of AS2676293 on RANKL-induced osteoclast differentiation in vitro. Images (**a**) and the number of TRAP-positive MNCs are shown (*n* = 3 samples per group) (**b**). Scale bar, 300 μm. **c**, **d** Representative histograms (**c**) and the average frequencies (**d**) of BrdU^+^ osteoclast precursor cells after stimulation with RANKL and M-CSF (*n* = 3 samples per group). **e** Frequencies of cell death (Annexin V^+^) in AS2676293-treated BMMs (*n* = 4 samples per group). *P < 0.05; **P < 0.01; ***P < 0.005; NS not significant. The data are presented as the means ± s.e.m.

AS2676293 was initially identified as a small-molecule chemical compound that potently inhibits RANKL-induced osteoclast differentiation of the murine monocyte/macrophage cell line RAW264.7.^22^ However, the effect of AS2676293 on primary cells remains to be determined. We first evaluated the effect of AS2676293 on RANKL-induced osteoclastogenesis using a murine bone marrow cell culture system: BMMs were stimulated with macrophage colony-stimulating factor (M-CSF) and RANKL, and multinucleated cells (MNCs) positive for tartrate-resistant acid phosphatase (TRAP) were counted as osteoclasts. AS2676293 exerted a suppressive effect on osteoclast differentiation (Fig. 1a, b). AS2676293 failed to inhibit RANKL-induced activation of extracellular signal–regulated kinase (ERK), IκBα and c-Jun N-terminal kinase (JNK) but strongly suppressed the induction of *Nfatc1* and *Fos* mRNA expression (Supplemental Fig. 1a and b).

Next, we analysed the effect of AS2676293 on the proliferation and survival of osteoclast precursor cells using 5-bromo-29-deoxyuridine (BrdU) incorporation and Annexin V assays, respectively. The AS2676293 treatment did not affect M-CSF-dependent proliferation of precursor cells or the rate of apoptosis, at least at a concentration <0.3 μmol ·L^−^^1^ (Fig. 1c–e). Thus AS2676293 exerted an inhibitory effect on RANKL-induced osteoclast differentiation without affecting cell proliferation or survival.

### AS2676293 abrogates the skeletal tumour burden in an osteolytic breast cancer metastasis model

**Fig. 2:**
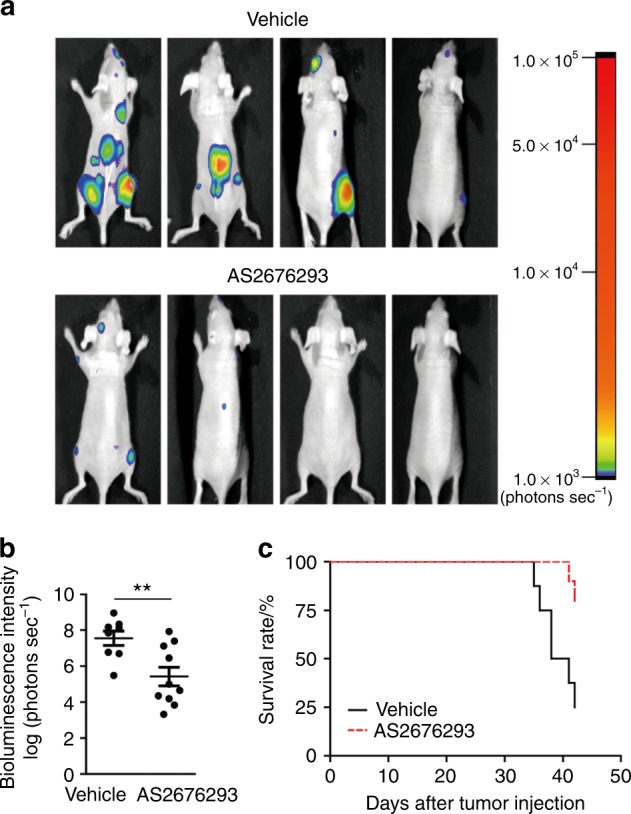
AS2676293 inhibits the bone metastasis of MDA-MB-231-5a-D-Luc2 cells. **a**, **b** Effect of AS2676293 on the bone metastasis of MDA-MB-231-5a-D-Luc2 cells. Representative bioluminescence images of mice (**a**) and quantification of the bioluminescence intensity on day 35 (vehicle, *n* = 8; AS2676293, *n* = 10) (**b**). **c** Survival rate of mice injected with MDA-MB-231-5a-D-Luc2 cells (vehicle, *n* = 8; AS2676293, *n* = 10). **P < 0.01. The data are presented as the means ± s.e.m.

We used a mouse model of osteolytic metastasis induced by the luciferase-labelled human breast cancer cell line MDA-MB-231-5a-D-luc2, a highly bone metastatic variant of MDA-MB-231 cells, to examine the effect of AS2676293 on bone metastasis.^24^ In this model, tumours spread into the bone marrow cavity, accompanied by the destruction of trabecular and cortical bone.^25,26^ Furthermore, the metastatic spinal cord compression results in hind limb paralysis, which seems to be the main cause of mortality. We performed an intracardial injection of MDA-MB-231-5a-D-luc2 cells into the nude mice, followed by daily oral administration of AS2676293 or vehicle. The distribution and development of the tumour were monitored and quantified using bioluminescence imaging. In most of the vehicle-treated mice, metastatic foci were detected in the skeletal tissues 35 days after injection (Fig. 2a). In contrast, AS2676293 administration potently inhibited the tumour burden in the skeletal tissues (Fig. 2a, b). Furthermore, AS2676293 administration effectively delayed the onset of tumour-induced death (Fig. 2c).

**Fig. 3:**
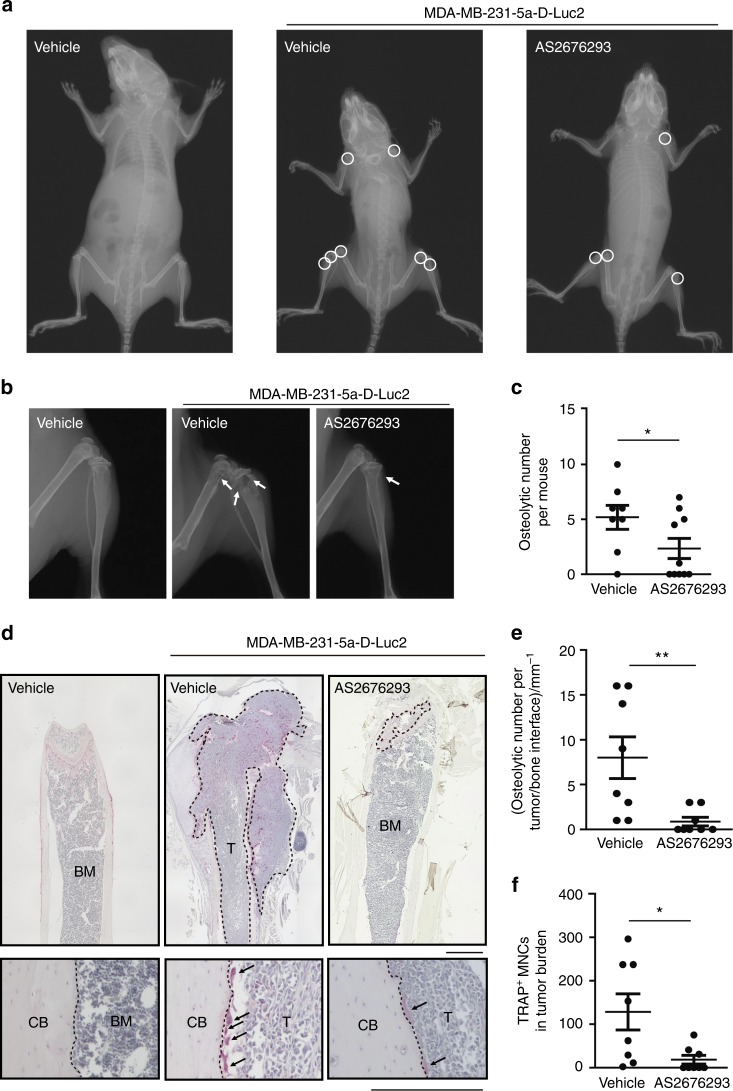
AS2676293 suppresses osteolysis and osteoclast activation in MDA-MB-231-5a-D-luc2 cell-bearing mice. **a**–**c** Effect of AS2676293 on osteolysis in MDA-MB-231-5a-D-luc2 cell-bearing mice. Representative soft X-ray images of mice (**a**) and hind limbs (**b**). The white circles and arrows indicate osteolytic lesions. Quantification of the number of osteolytic lesions per mouse (vehicle, *n* = 8; AS2676293, *n* = 10) (**c**). **d**–**f** Effect of AS2676293 on tumour-induced osteoclast formation. Representative images of TRAP-stained sections of the metaphysis of the distal femur (**d**). The area inside the dotted line in the upper panels delineates the tumour area. The dotted line in the lower panels indicates the cortical bone surface (T tumour, BM bone marrow, CB cortical bone). Upper scale bar, 1 mm; lower scale bar, 250 μm. The number of osteoclasts per tumour/bone interface (*n* = 8 per group) (**e**) and the number of TRAP^+^ MNCs in the tumour area (*n* = 8 per group) (**f**). *P < 0.05; **P < 0.01. The data are presented as the means ± s.e.m.

Next, tumour-induced osteolysis in MDA-MB-231-5a-D-Luc2-bearing mice was assessed using a soft X-ray analysis. We mainly observed osteolytic lesions in the humerus, scapula, femur, tibia and spine of vehicle-treated mice (Fig. 3a and Supplemental Fig. 2a). In particular, multiple osteolytic lesions were frequently observed in the metaphysis of the proximal tibia and distal femur of vehicle-treated mice (Fig. 3b). The administration of AS2676293 significantly inhibited the number of osteolytic lesions (Fig. 3a–c).

We examined whether tumour-induced osteoclast formation was inhibited by AS2676293 in vivo. We detected a substantial number of TRAP^+^ MNCs within the tumour tissues, particularly on the distal metaphyseal side of the femur in the vehicle-treated mice (Fig. 3d). Furthermore, consistent with previous reports,^25,26^ tumour-bearing mice exhibited an increase in the number of osteoclasts compared with tumour-free mice (Fig. 3d). In contrast, the administration of AS2676293 strikingly reduced the number of osteoclasts not only within the tumour areas (Fig. 3d, f) but also at the tumour/bone interface (Fig. 3d, e). In addition, the serum level of C-telopeptide of type I collagen (CTX) was decreased by the AS2676293 treatment (Supplemental Fig. 2b). Taken together, AS2676293 effectively inhibited tumour-induced osteoclast differentiation and the tumour burden in MDA-MB-231-5a-D-luc2 cell-bearing mice.

### AS2676293 inhibits the bone metastasis of B16F10-ZsGreen cells without affecting osteoclast activation

**Fig. 4:**
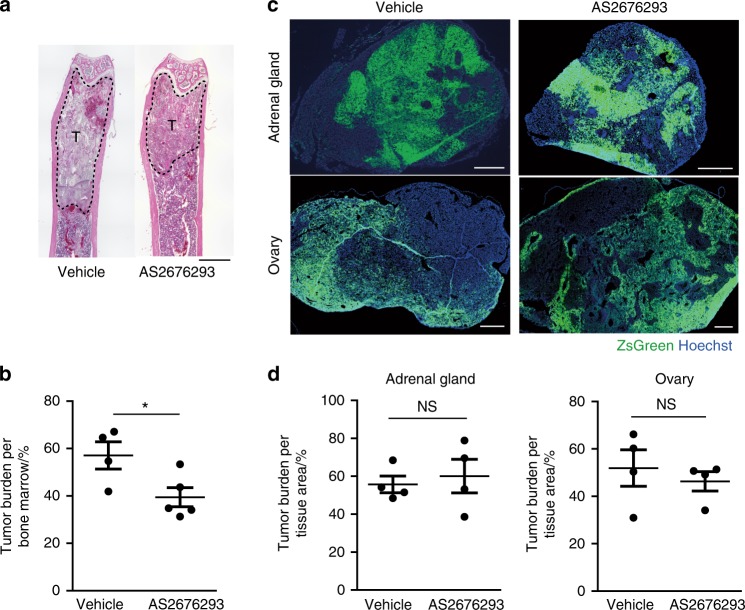
AS2676293 administration reduces the bone metastasis of B16F10-ZsGreen cells. **a**, **b** Effect of AS2676293 on the bone metastasis of B16F10-ZsGreen cells. Representative images of HE-stained femur sections (**a**). The area inside the dotted line indicates the tumour area. Scale bar, 1 mm. Histological quantification of the tumour burden (vehicle, *n* = 4; AS2676293, *n* = 5) (**b**). **c**, **d** Effect of AS2676293 on the adrenal and ovarian metastasis of B16F10-ZsGreen cells. Representative images of sections of the adrenal glands and ovaries (**c**). Scale bar, 500 μm. Histological quantification of the tumour burden (*n* = 4 animals per group) (**d**). *P < 0.05; NS not significant. The data are presented as the means ± s.e.m.

We next analysed a mouse model of metastasis using the murine melanoma cell line B16F10, which does not lead to osteoclast activation, to examine whether AS2676293 exerted its antitumour effect in an osteoclast-independent manner.^15^ The intracardial injection of B16F10 cells into syngeneic C57BL/6 mice invariably results in rapid metastasis to the skeleton.^15,27^ B16F10 cells also have the capacity to metastasize to non-skeletal tissues, such as the adrenal glands and ovaries.^15^ Since we were unable to easily distinguish the boundaries between tumour cells and soft tissues using haematoxylin–eosin (HE)-stained histological sections, we generated bright green fluorescence protein ZsGreen-expressing B16F10 cells (B16F10-ZsGreen) to accurately verify the tumour burden in the adrenal glands and ovaries. One day after the injection of B16F10-ZsGreen cells into syngeneic C57BL/6 mice, oral administration of AS2676293 was initiated and repeated every other day. AS2676293 administration significantly inhibited the tumour burden in the femur (Fig. 4a, b). B16F10-ZsGreen cells metastasize not only to the femur but also to other skeletal tissues.^27^ We observed black metastatic foci in the spine and cranium of vehicle-treated mice as a result of melanin pigment production (Supplemental Fig. 3a and b). In contrast, the administration of AS2676293 completely inhibited tumour foci formation in the cranium (Supplemental Fig. 3a and c). The number of tumour foci in the spine was also significantly decreased by AS2676293 administration (Supplemental Fig. 3b and c). In contrast to the antitumour effect of AS2676293 on bone metastasis, significant differences in the tumour burden or metastasis of B16F10-ZsGreen cells into the adrenal glands and ovaries were not observed between the vehicle and AS2676293 treatments (Fig. 4c, d). Based on these results, AS2676293 selectively inhibited bone metastasis and the skeletal tumour burden of B16F10 melanoma cells in bones.

**Fig. 5:**
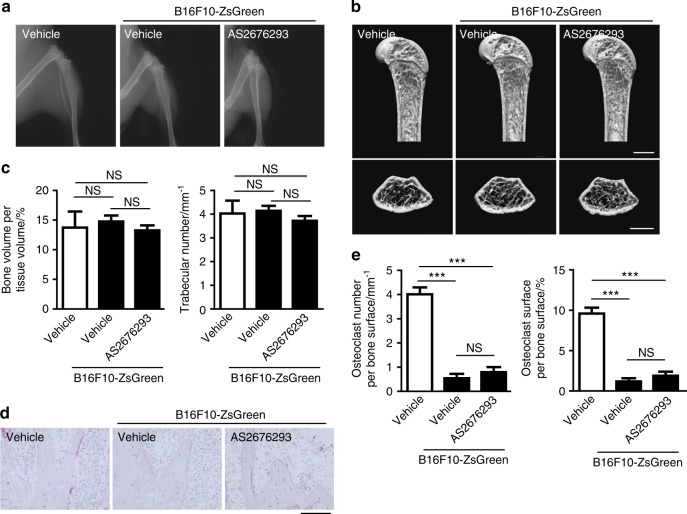
AS2676293 has no effect on bone mass or the osteoclast number in animals displaying bone metastasis of B16F10-ZsGreen cells. **a**–**c** Effect of AS2676293 on the bone mass of B16F10-ZsGreen cell-bearing mice. Representative soft X-ray images of hind limbs (**a**) and μCT of the femur (**b**). Scale bar, 1 mm. The bone volume and trabecular number were determined by μCT analysis (vehicle, *n* = 6; B16F10-ZsGreen cells with vehicle, *n* = 4; B16F10-ZsGreen cells with AS2676293, *n* = 6) (**c**). **d**, **e** Effect of AS2676293 on osteoclast formation in B16F10-ZsGreen cell-bearing mice. Representative images of TRAP-stained sections of the metaphysis of the distal tibia (**d**). Scale bar, 200 μm. Parameters for osteoclast number per bone surface and osteoclast surface per bone surface are shown (vehicle, *n* = 6; B16F10-ZsGreen cells with vehicle, *n* = 4; B16F10-ZsGreen cells with AS2676293, *n* = 6) (**e**). ***P < 0.005; NS not significant. The data are presented as the means ± s.e.m.

Consistent with previous reports,^15^ B16F10-ZsGreen tumour-bearing mice displayed neither osteolysis (Fig. 5a) nor bone mass reduction (Fig. 5b, c). Furthermore, a significant difference in bone volume was not observed between the AS2676293-treated and vehicle-treated mice (Fig. 5c). The bone morphometric analysis revealed a striking decrease in the number of osteoclasts induced by the bone metastasis of B16F10-ZsGreen melanoma cells (Fig. 5d, e). The administration of AS2676293 did not exert any additional inhibitory effect on osteoclast activity (Fig. 5d, e). Thus AS2676293 inhibited the bone metastasis of B16F10-ZsGreen melanoma cells without affecting osteoclastic bone resorption.

### AS2676293 exerts an inhibitory effect on RANKL-induced tumour cell migration

**Fig. 6:**
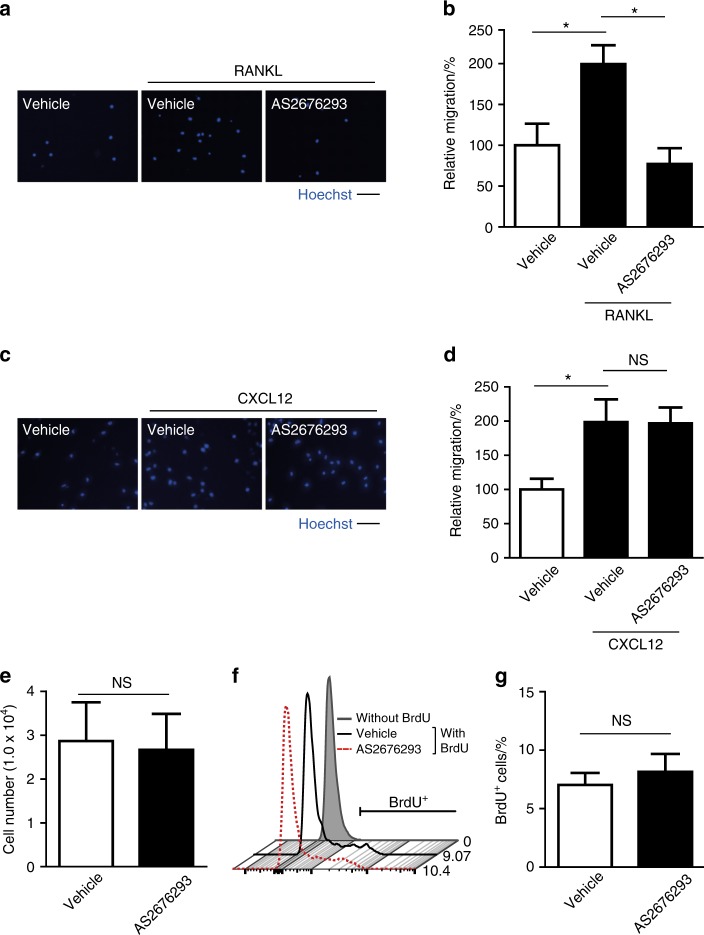
AS2676293 inhibits RANKL-mediated migration of B16F10-ZsGreen cells. **a**, **b** Effect of AS2676293 on RANKL-induced cell migration. Representative images of nuclear staining of B16F10-ZsGreen cells that migrated to the lower chamber containing medium with or without RANKL in the transwell migration assay (**a**). Scale bar, 100 μm. Percentage of B16F10-ZsGreen cells that migrated towards RANKL (normalized to vehicle-treated cells without RANKL as 100%, *n* = 4 per group) (**b**). **c**, **d** AS2676293 did not alter CXCL12-induced cell migration. Representative images of nuclear staining of B16F10-ZsGreen cells that migrated to the lower chamber containing medium with or without CXCL12 (**c**). Scale bar, 100 μm. Percentage of B16F10-ZsGreen cells that migrated towards CXCL12 (normalized to vehicle-treated cells without CXCL12 as 100%, *n* = 8 samples per group) (**d**). **e** The number of B16F10-ZsGreen cells after culture with or without AS2676293 for 16 h (*n* = 3 per group). **f**, **g** Effect of AS2676293 on the proliferation of B16F10-ZsGreen cells. Representative histograms (**f**) and the average frequencies (**g**) of BrdU^+^ B16F10-ZsGreen cells (*n* = 3 per group). *P < 0.05; NS not significant. The data are presented as the means ± s.e.m.

The aforementioned results prompted us to test whether AS2676293 blocks RANKL-induced chemotaxis of B16F10 melanoma cells using a Transwell migration assay. B16F10-ZsGreen cells plated in the upper chamber migrated through the membrane to the bottom chamber containing the chemotactic factor. A significant number of B16F10-ZsGreen melanoma cells migrated towards recombinant RANKL, which was completely blocked by the AS2676293 treatment (Fig. 6a, b). The chemokine C-X-C motif chemokine ligand 12 (CXCL12) also plays a crucial role in the targeting of bone metastatic cancer cells expressing its receptor CXCR4, including B16F10 cells.^15,28^ CXCL12-mediated migration of B16F10-ZsGreen cells was not abrogated by the AS2676293 treatment (Fig. 6c, d). Furthermore, treatment of B16F10-ZsGreen cells with AS2676293 did not exert significant effects on the number of B16F10-ZsGreen cells (Fig. 6e) or BrdU incorporation (Fig. 6f, g). Based on these results, AS2676293 has the capacity to prevent RANKL-mediated migration of B16F10 cells without affecting cell viability or proliferation.

## Discussion

According to the results from the present study, AS2676293 has the ability to block both RANKL-induced osteoclast differentiation of murine bone marrow cells and RANKL-mediated chemotaxis of tumour cells in vitro. Oral administration of AS2676293 effectively inhibited the bone metastasis of tumour cells not only by inhibiting osteoclastic bone resorption but also by suppressing tumour migration to bone. Thus a small-molecule compound targeting RANKL is a potential therapeutic treatment for bone metastasis.

AS2676293 suppressed osteoclast differentiation in vitro. AS2676293 potently inhibited RANKL-induced expression of *Fos* and *Nfatc1* during osteoclastogenesis, whereas it had no effect on RANKL-induced ERK, IκBα and JNK phosphorylation (Supplementary Figure 1a and b). Based on these results, AS2676293 targets the signalling pathway required for the induction of *Nfatc1* and *Fos* mRNA expression without affecting the activation of ERK, IκBα or JNK.

Denosumab has been approved as a treatment to prevent skeletal-related events in patients with bone metastasis. Although direct comparisons of the efficacy of AS2676293 with denosumab are difficult, AS2676293 inhibited bone metastasis of MDA-MB-231 cells more efficiently than OPG-Fc in a previous study.^14^ The excess bone resorption induced by tumour cells metastasizing to skeletal tissues causes osteolysis, which in turn accelerates tumour growth by amplifying a vicious cycle. Thus pharmacological inhibition of osteoclastic bone resorption is one of the main strategies for combatting bone metastasis. Bisphosphonate has been approved as a treatment for bone metastatic solid tumours.^7^ However, bisphosphonate exerted a significantly lower effect on the number of osteoclasts than an OPG treatment in C-26 colon carcinoma tumour-bearing mice, since the action of bisphosphonate is mainly limited to mature osteoclasts.^29^ As supported by the results that tumour-induced osteoclast activation was almost completely inhibited by AS2676293 administration (Fig. 3d–f), RANKL inhibition by a small-molecule compound and the established neutralizing antibody may serve as a promising strategy for osteolytic bone metastases. Although B16F10 cells did not stimulate osteoclast activation in a recent study, the authors did not clearly determine whether osteoclasts were unaffected or inhibited.^15^ Our study clearly showed that the bone metastasis of B16F10 cells inhibited osteoclast differentiation (Fig. 5d, e).

AS2676293 did not exert any additional effects on the decreased number of osteoclasts in the B16F10 metastasis models (Fig. 5d, e), probably because AS2676293 exerts both positive and negative effects on osteoclasts in this situation. AS2676293 not only directly inhibits RANKL-mediated osteoclastogenesis but also reduces bone metastasis-induced suppression of osteoclasts. Non-skeletal tumours were recently shown to act remotely on the bone tissues, even in the absence of local metastasis.^30^ Since AS2676293 did not affect tumour metastasis to the ovary or adrenal gland, it is also possible that tumours in other organs remotely affected the bone tissue, reducing the number of osteoclasts.

AS2676293 failed to completely suppress the bone metastasis of B16F10 cells. Other chemotactic factors, such as CXCL10, CXCL12 and osteopontin, are known to be associated with tumour migration.^10,31^ These factors may also contribute to the bone-specific migration of B16F10-ZsGreen cells.

Increasing evidence supports the importance of RANKL signalling in breast cancer development.^32,33^ Hormone replacement therapy increases the risk of developing breast cancer.^34,35^ Sex hormone-induced RANKL expression on mammary epithelial cells causes the expansion of RANK^+^ mammary progenitor cells, which contributes to tumorigenesis.^33,36,37^ RANKL inhibition prevents the development of mammary cancer induced by the progesterone derivative medroxyprogesterone acetate in mice.^37^ Recently, the RANKL–RANK pathway was shown to play a key role in breast cancer driven by mutations in the *breast cancer* (*BRCA*) 1.^38,39^ Therefore, small-molecule compounds targeting RANKL signalling may represent a useful strategy for preventing breast cancer in carriers of *BRCA1* mutations.

Since RANKL–RANK signalling is involved in not only autoimmune responses but also immune tolerance, a precise estimation of the impact of RANKL inhibition on antitumour immunity is difficult to determine.^1^ However, according to recent clinical and animal studies, RANKL inhibition in combination with immune checkpoint blockade enhances antitumour immune responses. Clinical case reports have suggested the efficacy of a combination treatment with an anti-RANKL antibody and anti-cytotoxic T-lymphocyte-associated protein 4 (anti-CTLA-4) antibody in patients with melanoma presenting with bone metastasis.^40^ In the pulmonary metastasis model of B16F10 cells, an increase in T cell infiltration into tumours was observed following the administration of a combination of anti-RANKL and anti-CTLA-4 antibodies but not the anti-CTLA-4 antibody alone.^40,41^ Therefore, the administration of AS2676293 in combination with immune checkpoint inhibitors would be expected to exert more potent antitumour effects on bone metastasis. Orally available medications targeting the RANKL signalling pathway should prove beneficial in reducing the patient’s tumour burden, thus providing an attractive alternative approach for the treatment of tumour metastasis.

## Materials and methods

### Mice

C57BL/6 and BALB/c nu/nu mice were purchased from Clea Japan, Inc. All animal experiments were performed with the approval of the Animal Ethics Committee of The University of Tokyo and conducted in accordance with institutional guidelines.

### Bone metastasis model of MDA-MB-231-5a-D-Luc2 cells

MDA-MB-231-5a-D-Luc2 breast cancer cells, a highly bone metastatic variant of human breast cancer MDA-MB-231 cells,^42^ were maintained in culture in Dulbecco’s modified Eagle’s medium (DMEM; GIBCO) supplemented with 10% foetal calf serum at 37 °C with a 5% CO_2_ atmosphere. The harvested cell suspension was washed twice with phosphate-buffered saline (PBS) and resuspended at 4 °C immediately prior to injection. MDA-MB-231-5a-D-Luc2 cells (1.0 × 10^6^ cells) were suspended in 0.2 mL of PBS and injected into the left ventricle of 4-week-old BALB/c nu/nu mice. The administration of AS2676293 (10 mg· kg^−1^) was repeated daily starting 1 day after the tumour cell injection. Mice injected with an equal volume of 0.5% methylcellulose were analysed as controls. In vivo bioluminescence imaging and enzyme-linked immunosorbent assay (ELISA) of CTX in serum were performed on day 35 after the injection. Histological and soft X-ray analyses were performed on day 42 or immediately after death for mice that did not survive until day 42.

### Bone metastasis model using B16F10-ZsGreen cells

The murine melanoma cell line B16F10 was purchased from ATCC. ZsGreen-expressing B16F10 cells (called B16F10-ZsGreen) were generated by retroviral transduction with pSIREN-RetroQ-ZsGreen (Clontech). B16F10-ZsGreen cells (5.0 × 10^5^ cells) were injected into the left ventricle of 8-week-old female C57BL/6 mice. The administration of AS2676293 (50 mg· kg^−1^) was repeated every other day starting 1 day after the injection of B16F10-ZsGreen cells. Histological and microcomputed tomography (μCT) analyses were performed on day 12.

### μCT analysis

The right femur was subjected to μCT analysis. Scanning was performed using a ScanXmate-A100S Scanner (Comscantechno), as previously described.^43,44^ Three-dimensional microstructural images were reconstructed, and structural indices were calculated using TRI/3D-BON software (RATOC).

### Soft X-ray analysis

Soft X-ray imaging was performed using a CMB-2 X-ray irradiation apparatus (SOFTEX). Mice were placed on X-ray films (FUJIFILM) and exposed to X-irradiation at 55 kV and 25 mA for 2 s. The irradiated films were developed using a Kodak X-Omat Processor (Kodak).

### In vivo bioluminescence imaging

The tumour burden was quantified using whole-body in vivo bioluminescence imaging with a NightOWL LB981 system (Berthold Technologies). Mice were intraperitoneally injected with 2.5 mg of D-luciferin potassium salt (Promega) dissolved in 0.2 mL of PBS. Images were acquired beginning 10 min after D-luciferin injection. Regions of interest (ROIs) were drawn around the whole body. The photons emitted from the ROIs was quantified in units of photons per second using the IndiGO software (Berthold Technologies).

### ELISA

Mice were fasted for 6 h prior to the analysis of serum CTX levels. Blood was collected by cardiac puncture. Serum was isolated by centrifugation at 10 000 revolutions min^−^^1^ for 10 min. Quantification was performed using the RatLap EIA Kit from IDS (Immunodiagnostic Systems) according to the manufacturer’s instructions.

### Histological analysis

For paraffin sections, tissues were fixed with 4% paraformaldehyde, decalcified with OSTEOSOFT (Merck Millipore) at 4 °C for 3 weeks and then embedded in paraffin after dehydration. Paraffin blocks were cut into 7-μm-thick sections. HE staining was performed by staining sections with haematoxylin (Muto Pure Chemicals) for 5 min, followed by 30 s of staining with eosin (Wako). We evaluated the tumour burden in three coronal sections of the femur from each mouse (21-μm intervals between consecutive sections). The bone marrow area occupied by tumour cells was measured using a BZ-II Analyser (Keyence). TRAP staining was performed at room temperature for 5 min, followed by nuclear counterstaining with haematoxylin. Static parameters of bone resorption were measured in a defined area ranging in distance from 300 to 1 200 μm from the growth plate using an OsteoMeasure bone histomorphometry system (Osteometrics).

For cryosections, tissues were fixed with 4% paraformaldehyde overnight at 4 °C. Samples were washed twice with PBS, incubated with a 30% sucrose solution in PBS overnight and then embedded in optimum cutting temperature compound (Sakura Finetek). Frozen blocks were cut into 5-μm-thick sections. Slides were first hydrated for 5 min and then permeabilized with 0.2% Triton X-100 in PBS for 20 min. Slides were subsequently stained with 0.1% Hoechst in PBS for 10 min and mounted with Fluorescent Mounting Medium (DAKO).

### In vitro osteoclast differentiation

In vitro osteoclast differentiation was performed using previously described methods.^45,46^ Bone marrow cells were isolated from the mouse femur.using PBS. Bone marrow cells were seeded in 24-well plates (BD Falcon) and cultured in α-Minimum Essential Medium (α-MEM) (Invitrogen) supplemented with 10% foetal bovine serum (FBS) and M-CSF (10 ng· mL^−1^) (R&D Systems) for 2 days to obtain BMMs. After 2 days of incubation, BMMs were further cultured in α-MEM containing 10% FBS, M-CSF (10 ng· mL^−1^) and RANKL (50 or 5 ng ·mL^−1^) with or without AS2676293. Osteoclasts were fixed 72 h after RANKL stimulation and stained for TRAP. The number of TRAP-positive MNCs containing more than three nuclei in each well (growth area per well, 2.0 cm^2^) was counted. Apoptosis was assayed 24 h after RANKL stimulation using an Apoptotic/Necrotic/Healthy Cells Detection Kit (PromoKine). Photographs of the stained cells were captured with a fluorescence microscope BZ-9000E (Keyence), and the cell number was counted using the BZ-II analysis software (Keyence). Total RNA was extracted with ISOGEN (NIPPON GENE) according to the manufacturer’s instructions. First-strand complementary DNAs were synthesized using Superscript III reverse transcriptase (Invitrogen). Quantitative reverse transcriptase-PCR analyses were performed with the LightCycler apparatus (Roche Applied Science) using SYBR Green Real-time PCR Master Mix (TOYOBO). The level of mRNA expression was normalized to the *Gapdh* mRNA. The following primers were used: *Gapdh*, 5’-ACCCAGAAGACTGTGGATGG-3’ (sense) and 5’-CACATTGGGGGTAGGAACAC-3’ (antisense); *Nfatc1*, 5’-TTCCTTCAGCCAATCATCCCCCCAGTTAC-3’ (sense) and 5’-CGATGTCTGTCTCCCCTTTCCTCAGCTC-3’ (antisense); and *Fos*, 5’-GGGACAGCCTTTCCTACTACC-3’ (sense) and 5’-GATCTGCGCAAAAGTCCTGT-3’ (antisense).

### Transwell migration assay

The cell migration assay was performed using a polycarbonate membrane polystyrene insert (3422, Corning) with an 8-µm pore size in 24-well dishes. The polycarbonate membrane was precoated with fibronectin (10 μg· mL^−1^) for 2 h. After cells were cultured in serum-free DMEM for 12 h, 2 × 10^4^ cells in 200 µL of serum-free DMEM supplemented with 0.1% bovine serum albumin and 12 mmol·L^−1^ HEPES were plated in the upper chamber. Serum-free DMEM with or without RANKL (100 ng· mL^−1^) or CXCL12 (100 ng· mL^−1^) was placed in the lower chamber. AS2676293 (0.3 μmol· L^−1^) was added to the upper chamber. After an incubation for 16 h, cells on the upper side of the membrane were removed with a cotton applicator. Cells that had migrated to the lower surface of the membrane were fixed with 4% paraformaldehyde for 20 min and stained with Hoechst for 10 min. Micrographs of the migrated cells were captured using a fluorescence microscope BZ-9000E (Keyence), and the cell number was counted with the BZ-II analysis software (Keyence).

### In vitro BrdU incorporation assay

An in vitro BrdU incorporation assay was performed using a FITC BrdU Flow Kit (BD Biosciences) according to the manufacturer’s instructions. BMMs were stimulated with RANKL (100 ng· mL^−1^) and M-CSF (10 ng· mL^−1^) in the absence or presence of AS2676293 for 16 h to determine the proliferation rate of osteoclast precursor cells. For the analysis of tumour cell proliferation, B16F10-ZsGreen cells were stimulated with or without AS2676293 (0.3 μmol· L^−1^) for 16 h. The incorporated BrdU was evaluated using a FACSCantoII flow cytometer (BD Biosciences).

### Immunoblot analysis

BMMs were serum-starved for 6 h, and then cell lysates were harvested at the indicated times after RANKL stimulation (50 ng· mL^−1^). Immunoblot analyses were performed using specific antibodies against ERK (9102, Cell Signaling Technology), phospho-ERK (9101S, Cell Signaling Technology), JNK (9252S, Cell Signaling Technology), phospho-JNK (9255S, Cell Signaling Technology), IκBα (9242S, Cell Signaling Technology), phospho-IκBα (9241S, Cell Signaling Technology) and β-actin (AC-15, Sigma) as the primary antibodies; horseradish peroxidase-conjugated anti-mouse IgG (GE Healthcare) and anti-rabbit IgG (Cell Signaling) as the secondary antibodies; and the ECL Plus Western Blotting Detection reagents (GE Healthcare) for detection, according to the manufacturers’ instruction.

### Statistical analyses

We performed statistical analyses using Student’s *t* test or analysis of variance (ANOVA) followed by Dunnett’s or Tukey’s (for one-way ANOVA) multiple comparison test (*P < 0.05; **P < 0.01; ***P < 0.005; NS, not significant). The statistical analysis of the survival rate was performed using log-rank test. All data are reported as means ± s.e.m.

## Electronic supplementary material

Supplemental Figure 1

Supplemental Figure 2

Supplemental Figure 3

Supplemental Figure 4
