# Supplementary figures and images for: Efficacy of an orally active small-molecule inhibitor of RANKL in bone metastasis
Source: Bone Res. 2019 Jan 3;7:1. doi: 10.1038/s41413-018-0036-5 (PMC6315020; doi:10.1038/s41413-018-0036-5)

Supplemental Figure 1

a

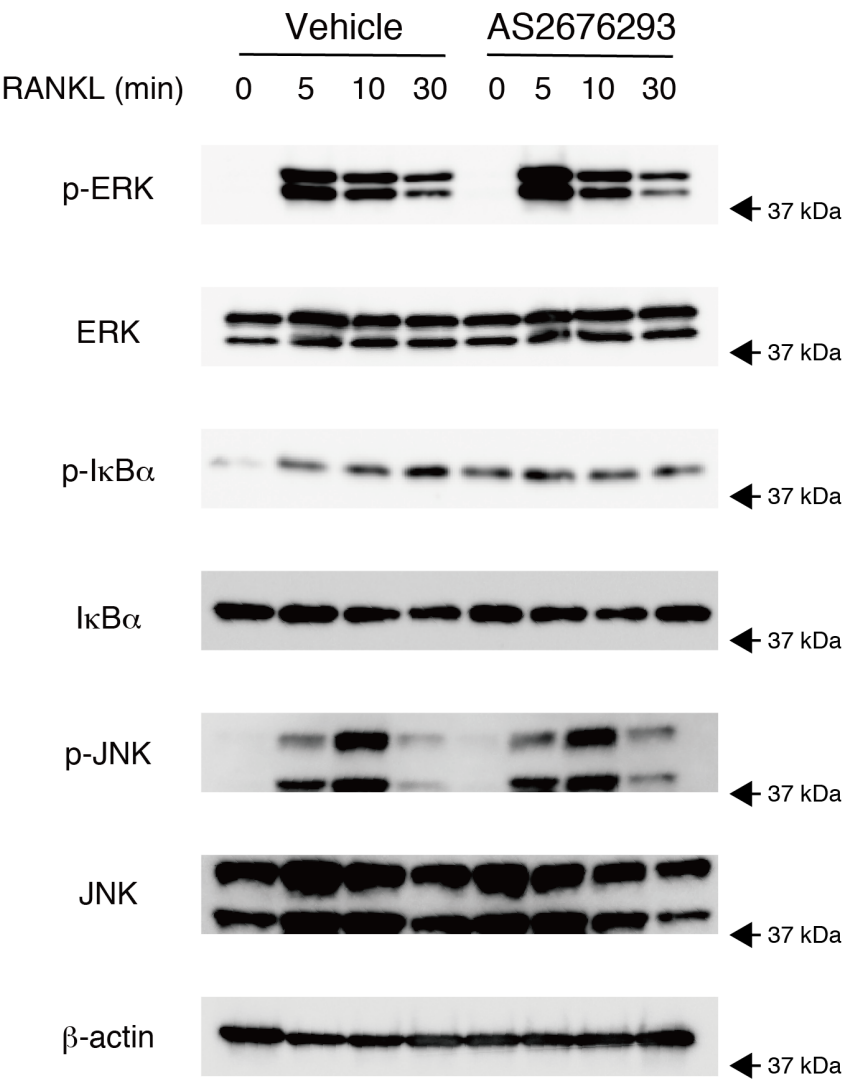

b

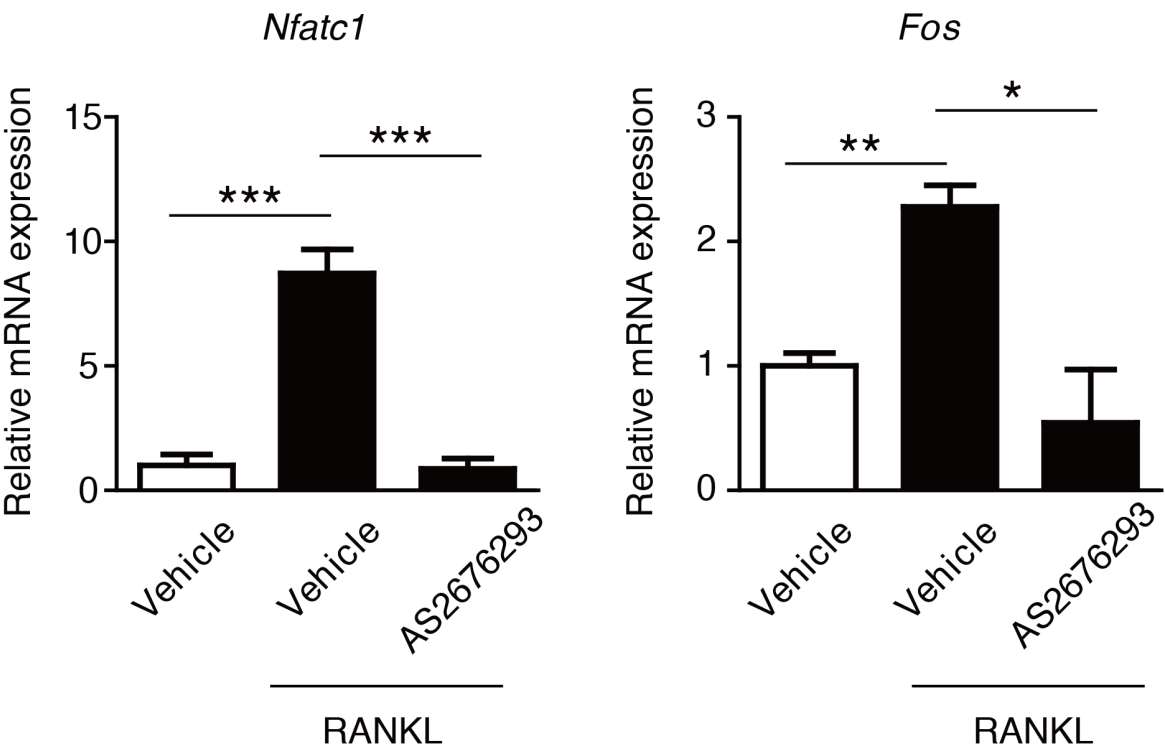

Supplement: Supplementary file 1 — Supplemental Figure 1 [file 41413_2018_36_MOESM1_ESM.pdf]

Supplemental Figure 2

**a**

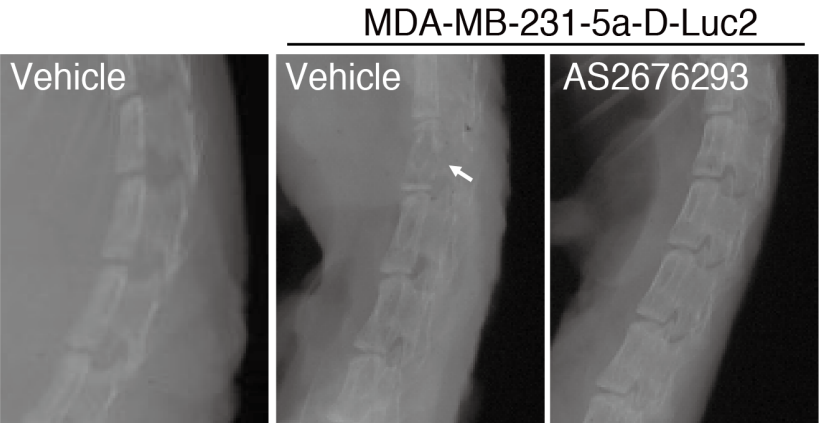

**b**

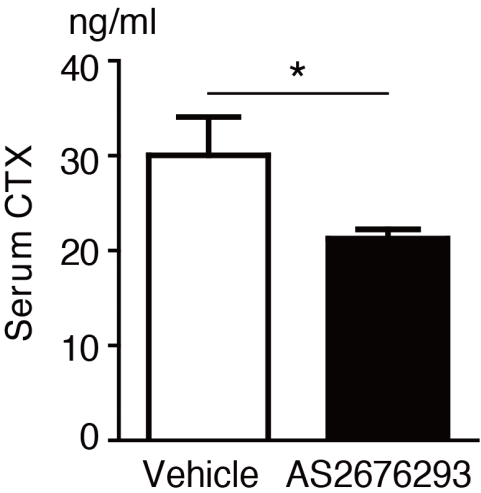

Supplement: Supplementary file 2 — Supplemental Figure 2 [file 41413_2018_36_MOESM2_ESM.pdf]

Supplemental Figure 3

a

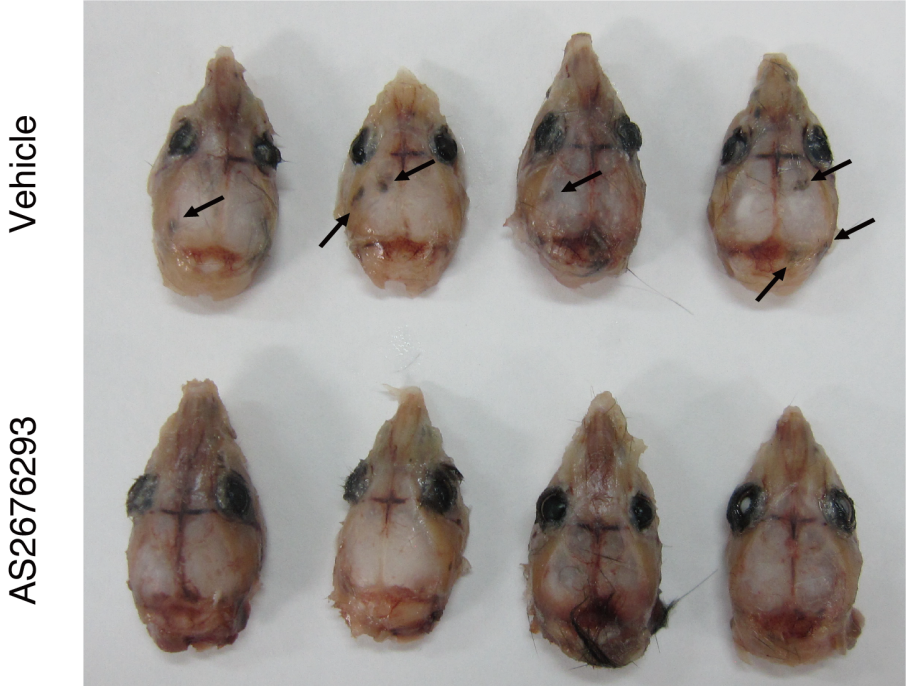

b

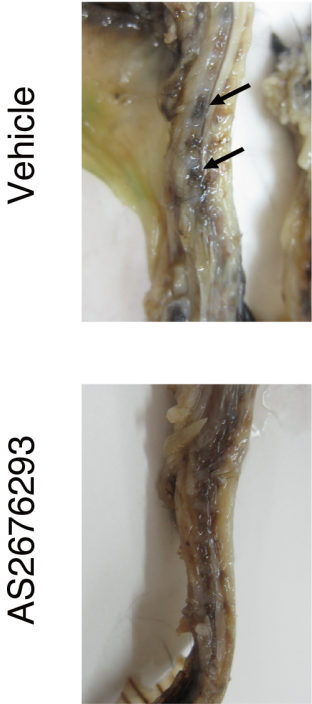

c

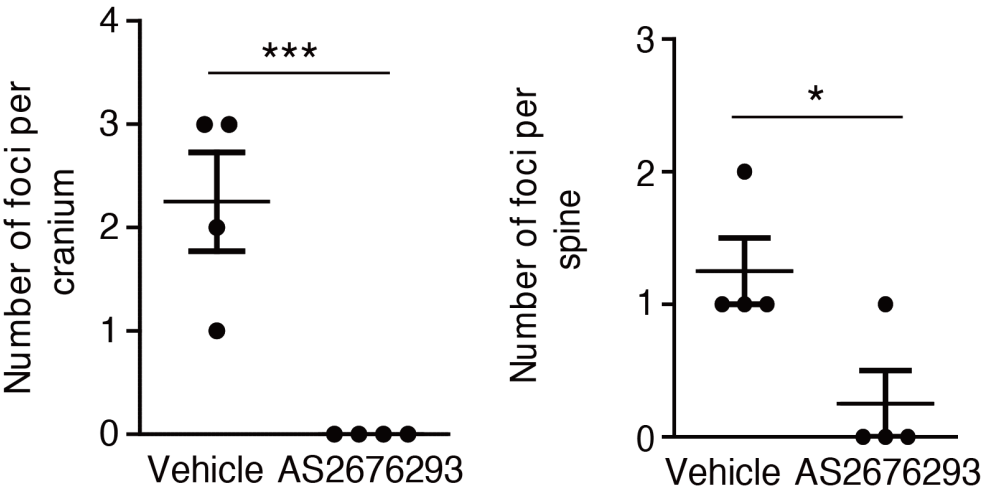

Supplement: Supplementary file 3 — Supplemental Figure 3 [file 41413_2018_36_MOESM3_ESM.pdf]
