# Supplementary material for: Efficacy of an orally active small-molecule inhibitor of RANKL in bone metastasis
Source: Bone Res. 2019 Jan 3;7:1. doi: 10.1038/s41413-018-0036-5 (PMC6315020; doi:10.1038/s41413-018-0036-5)
Supplement: Supplementary file 4 — Supplemental Figure 4 [file 41413_2018_36_MOESM4_ESM.docx]

**Supplemental Figure 1**

**Effects of AS2676293 on *Nfatc1* and *Fos* expression and activation of signalling intermediates after RANKL stimulation**

(**a**) RANKL-induced phosphorylation of ERK, JNK and IκBα in BMMs treated with vehicle or AS2676293. (**b**) Expression of *Nfatc1* and *Fos* in vehicle- or AS2676293-treated BMMs 3 days after RANKL (5 ng ml^-1^) stimulation (N=3 samples per group). *P < 0.05; **P < 0.01; ***P < 0.005. The data are presented as the means ± s.e.m

**Supplemental Figure 2**

**AS2676293 administration reduces tumour-induced osteolysis**

(**a**) Effect of AS2676293 on osteolysis in MDA-MB-231-5a-D-luc2 cell-bearing mice. Representative radiographs of the spine. Arrow shows osteolytic lesions. (**b**) Serum CTX levels (vehicle, N=6; AS2676293, N=5). *P < 0.05. The data are presented as the means ± s.e.m.

**Supplemental Figure 3**

**Administration of AS2676293 inhibits metastasis of B16F10-ZsGreen cells to the cranium and spine**

(**a-c**) Effect of AS2676293 on the metastasis of B16F10-ZsGreen cells to the cranium and spine. Representative images of the cranium and spine (**a and b**). Arrows show black metastatic foci. (**c**) The number of metastatic foci per cranium and spine (N=4 animals per group). ***P < 0.005; *P < 0.05. The data are presented as the means ± s.e.m.
